# Evolution at the arid extreme: the influence of climate on sand termite colonies and fairy circles of the Namib Desert

**DOI:** 10.1098/rstb.2022.0149

**Published:** 2023-08-28

**Authors:** Norbert Juergens, Alexander Groengroeft, Felicitas Gunter

**Affiliations:** ^1^ Institute of Plant Science and Microbiology, University of Hamburg, 22609 Hamburg, Germany; ^2^ Institute of Soil Science, University of Hamburg, 20146 Hamburg, Germany

**Keywords:** *Psammotermes*, Namib Desert, nests, social insects, fairy circles

## Abstract

In the hyperarid Namib Desert, the sand termite *Psammotermes allocerus* Silvestri, 1908 (Isoptera: Rhinotermitidae) establishes colonies that create conspicuous, barren patches known as ‘fairy circles' on permeable, sandy soils. The central bare areas of fairy circles serve the key function of storing moisture received from sparse rainfall. The sandy soil texture allows rapid infiltration and percolation of precipitation, while localized herbivory by the termites creates the bare patch, thereby reducing the rapid loss of soil moisture by the uptake and transpiration of water by plants. The resulting storage of rain water even during prolonged periods of drought enables perennial life in hyperarid desert environments and forms a globally unique example of ecosystem engineering by social insects. During the past decade, most publications primarily debated the origin of fairy circles. Here, we contribute to the special issue with a focus on the functional and evolutionary dimension of the structure of the *Psammotermes* colony with two differing nest types and two spatially separated key resources, as a successful adaptation to extreme desert environment. The paper is primarily a review and a synthesis of previous work, with the inclusion of new, relevant findings.

This article is part of the theme issue ‘The evolutionary ecology of nests: a cross-taxon approach’.

## 1. Introduction

The Namib Desert, home of the fairy circles, forms a relatively narrow band along the western coast of southern Africa extending from north of the Namibian–Angolan border and along the entire Atlantic coast of Namibia to the north-western tip of South Africa. Here, it merges with the arid to semiarid Succulent Karoo. Today, the Namib is one of the most arid regions in the world, receiving well less than an annual average of 10 mm precipitation in the most arid parts.

The Namib is also the desert with the longest history of uninterrupted aridity, at least since 15 MA [1,2]. Because of the long duration of aridity, the Namib Desert is rich in endemic plants and animals that have evolved unusual adaptations that allow survival the harsh, arid conditions, in some regions combined with high air humidity and even occurrence of fog. The ‘living stones’ (Aizoaceae) [3,4], the psammophorous plants [5], the tenebrionid beetles [6] and the ‘living fossil’ *Welwitschia mirabilis* [7,8] are only a few examples.

**Figure 1.**
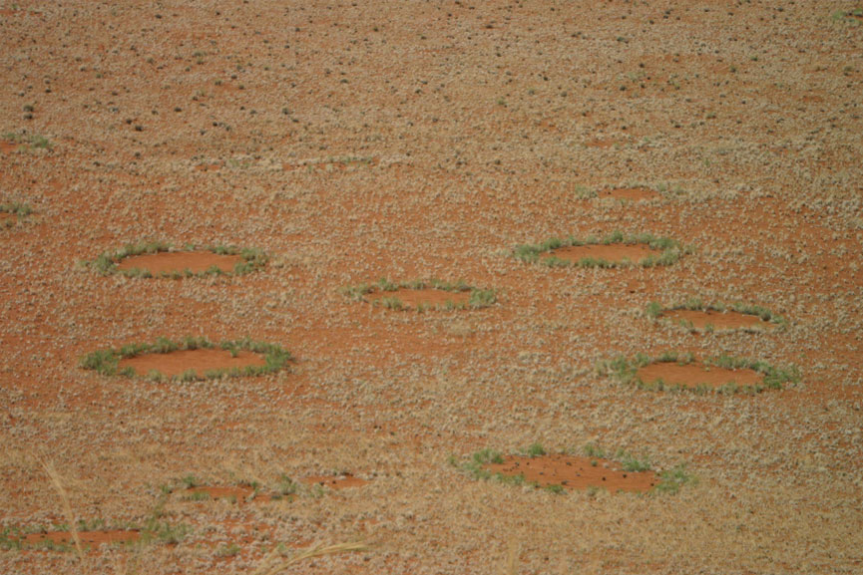
Fairy circles with about 4 m diameter, composed of a circular bare patch in the centre, a ring of tall green grass (*Stipagrostis ciliata*) that survives many years (perennial belt) and surrounding grassland (matrix) (also *Stipagrostis ciliata*) that had already died in the above photo. (Image credit: Sven-Eric Stender, 13 March 2008, Dieprivier, Gondwana Namib Park, Namibia.) (Online version in colour.)

One of the more unusual features of the Namib Desert are the ‘fairy circles’—circular patches of sandy soil that completely lack plant cover—regularly distributed within a sparsely vegetated landscape (figure 1). These bare patches (fairy circles) only occur on sandy soils with no more than 2% clay [9], and they have mean diameters ranging from more than 3 m in the south to more than 22 m in the north. Their density ranges from 1 to 74 per hectare and the average centre-to-centre nearest neighbour distances are mostly in the range between 10 and 20 m. The bare patch is typically surrounded by a ring (fairy ring) of tall long-lived grass tussocks (figure 1). Most fairy circles in the summer rainfall climate of Angola and most of Namibia form in sparse grassland. Only in the Succulent Karoo in the southwest of Namibia and in the Richtersveld of north-western South Africa is the vegetation around the bare circles is formed by woody dwarf shrubs with succulent leaves [10].

The first scientific documentation of fairy circles was published in 1971 [11], but only during the past decade have articles about fairy circles and scientific controversies about their origin repeatedly appeared both in scientific journals and the popular press. In the early days, fairy circles of the Namib, including those that occur in southwestern Angola, were regarded as a single phenomenon [12–15]. Later studies [16] have revealed that a different type of very large fairy circle is found only in the northernmost tip of the Namib Desert (Angola, north of the Rio Curoca), and it is associated with a newly discovered termite species of the family Hodotermitidae. Those colonies occur in a slightly more humid climate with up to 200 mm mean annual precipitation (MAP) [16]. This article will solely focus on the more arid fairy circles of the Namib Desert in Angola, Namibia and South Africa. These receive lowest MAP in the Succulent Karoo (50–80 mm MAP) and the Southern Namib (10–120 mm MAP), while the fairy circles in north-western Namibia and Angola are in the range from 70 to 150 mm MAP ([17], fig. 7.3.2).

Strong evidence points to the central role of the sand termite, *Psammotermes allocerus*, in the formation of fairy circles. In particular, the manner in which the termites locally eliminate vegetation from the circular patches functions to conserve soil moisture required for colony survival.

The goal of this paper is to highlight the association of nests of *Psammotermes* with fairy circles and the role that the termites play in the context of the vegetation and ecosystem function. The peculiar behaviour of the termites is apparently an adaptation to extreme desert conditions and is an example of ecosystem engineering by colonies of social insects. For this purpose, we present

(1) a brief review of the controversy on the origin of fairy circles and the evidence pointing to the role of sand termites;
(2) a look at water as the essential resource in hyperarid environments and how colonies of sand termites manage and secure limited and unpredictable moisture resources;
(3) details on the structure of colonies and nests of *Psammotermes*, and finally;
(4) consideration of the evolutionary responses of the termites to extreme aridity.

This paper is primarily a review and synthesis of previous published work, combined with additional new findings.

## 2. Controversy over the origin of fairy circles

Like the unusual mounds called *heuweltjies* found throughout the Succulent Karoo of western South Africa and southernmost Namibia (see paper on heuweltjies in this issue [18]), processes responsible for the formation of fairy circles in the Namib Desert have been long debated. We recently published a comprehensive review ([10], ch. 4). The four more frequently discussed hypotheses are briefly discussed below.

### (a) *Euphorbia* toxicity hypothesis

Theron [19] proposed that toxic substances from dead spurges, especially the large shrub *Euphorbia damarana*, suppress grass growth and create fairy circles. Recently, Meyer *et al.* [20,21] updated this hypothesis. They proposed that the adhesive, hydrophobic and toxic latex causes and maintains fairy circles. They also claimed that diameter and spatial patterns are similar to fairy circles. Getzin *et al.* [22] reject the *Euphorbia* toxicity hypothesis mainly because they found a mismatch in dimensions and state that ‘the size of dead *Euphorbias* cannot explain the size of observed FCs and the spatial distribution of *Euphorbias* cannot cause the specific pattern signature of FCs’. Here, we confirm that in 95% of more than 1700 recorded fairy circles the vegetation did not contain any *Euphorbia* species.

### (b) The geochemical gas hypothesis

Soils from the bare patches of fairy circles can inhibit the germination or growth of grasses [13,23,24]. Naude *et al.* [25] measured a wide range of semivolatile organic compounds in gas sampled from the soil of fairy circles and attributed the absence of plants to the effects of those compounds. They interpreted these gases as products from geochemical processes in deeper geological layers.

Jürgens [26] confirms the presence of the organic compounds from soils of fairy circles, but concluded that the gases are generated by the digestive systems of termites rather than by physical geological processes. However, in certain geological settings, natural seepages of hydrogen gas have been documented. For example, hydrogen seeps within large vegetation gaps up to 250 m in diameter have recently been reported in Namibian savannah and woodland vegetation [27]. Although these structures were referred to as ‘fairy circles’ in this report, they are distinctly different in size and also in geographical distribution, being found in areas with MAP of over 200 mm, rather than in the extremely arid environments of the Namib Desert where fairy circles occur. The use of the same term for such different phenomena unfortunately only adds to confusion regarding the origin of either.

### (c) Self-regulation hypothesis

Based on the early reaction–diffusion theory of morphogenesis by Turing [28] and the consequences of scale-dependent feedbacks [29], a theoretical foundation for processes in systems has been developed. These theoretical foundations have been used to explain a wide variety of spatial patterning of vegetation, ranging from fully vegetated to regularly spaced bare patches, to banding, to regularly spaced vegetation islands [30,31]. This school attempts to provide a universal explanation for vegetation patterning in arid to semiarid zones based exclusively on plant–plant interactions (the interplay between short-distance facilitation and long-distance competition). Numerous publications use the regularity of the spatial pattern of fairy circles as a starting point and argue that competition for water (and nutrients) results in rapid transport of water over several metres and ultimately causes the grass to die back in circular patterns [15,32,33].

Tarnita *et al.* [34] demonstrate theoretically (with direct comparisons to the Namib Desert fairy circles) that the kind of patterning that actually exists in this system in nature is predicted much more accurately by an approach that considers effects of neighbouring termite colonies along with plant–plant interactions. The conclusions of Tarnita *et al*. are, in general, that the large-scale pattern of the regularly spaced fairy circles is generated by interactions among the termite colonies, but the interactions among plants play out on a finer spatial scale in the vegetated zones outside of fairy circles.

The assumed water transport rates in sandy soils are an important issue. Cramer *et al.* [33] and Getzin *et al.* [35] assume water movements of many metres within a few days. By contrast, Gröngröft & Jürgens [9] and Jürgens & Gröngröft [36] provide detailed soil moisture measurements at different soil depths, sampled from 2008 until 2023. They also established the hydraulic properties of the fairy circle sand and provided evidence that the sands of the fairy circle landscapes have very low unsaturated hydraulic conductivities that do not allow water transport or feedbacks on the scale of metres in horizontal distance, as required by the hypothesis that fairy circles arise through a process of vegetation self-organization.

### (d) Sand termite hypothesis

There is broad evidence that indicates colonies of the sand termite (*Psammotermes allocerus* species complex) of the termite family Rhinotermitidae play an essential role in the formation of fairy circles of the Namib Desert region [14,37,38]. The most convincing evidence includes:

— In all landscapes containing fairy circles, from the Iona National Park in Angola to the Richtersveld in South Africa, colonies and activities of *Psammotermes* (living animals, nests, soil dumps, tunnels or galleries) have been observed within fairy circles. To date, the record includes 1799 fairy circles that have been examined in detail by Jürgens and collaborators.
— The mechanism directly responsible for the bare patch of fairy circles is the elimination of newly germinated seedlings by the termites [10,14,37]. After pulses of rainfall that trigger germination of seedlings, *Psammotermes* attacks and damages the root systems, causing the young plants to die.
— The termites of one fairy circle are members of a single, genetically identical colony [39].

## 3. Soil moisture as the limiting resource in a hyperarid environment controlled by sand termites

Before the water relations at *Psammotermes* colonies in fairy circles are explored, a short look at the water relations of other termite taxa in slightly more humid climates helps to underline the differences between these environments and termite behaviour. For example, in the arid savannah of Namibia, only 100 km east of the fairy circle belt, with MAP only 100 mm higher than in the fairy circle environments, *Macrotermes michaelseni* forms huge central nests containing fungus gardens within large, above-ground mounds [40,41]. The mass and architecture of the mounds play a functional role in regulation of temperature, moisture and gases within the space inhabited by the colony and fungus gardens [42–45]. The bodies of *Macrotermes* termites, as well as termites in general, are extremely vulnerable to dehydration. The large colonies of *Macrotermes* are capable of providing for their water needs by ‘mining’ for soil water stored at great depth via their extensive tunnel systems [46–49] and bringing moisture-laden soil up to the large colony and its fungus garden. The cemented exterior of the tall mound impedes water losses through evaporation and also provides protection from predators [50]. The large size of individual colonies of *Macrotermes* and the mounds is only possible because of the generally high levels of available resources, including water, in these more productive savannah environments [41,45,51,52].

**Figure 2.**
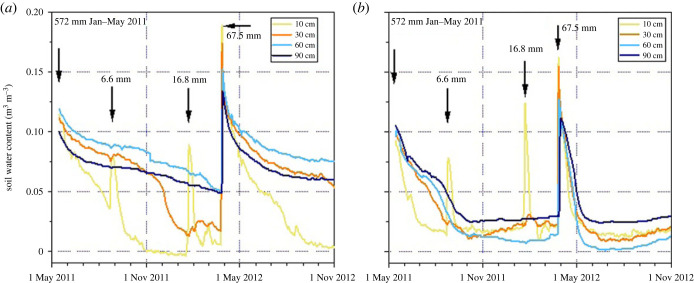
Course of water content in the soil beneath the bare patch of a fairy circle (*a*) and beneath the neighbouring matrix vegetation (*b*) at Dieprivier, Namib Desert Park, spanning the period from May 2011 to November 2012, measured with TDR sensors. Note the extreme amount of rain that fell before the shown period of time during January to May 2011, followed in the next year by a single strong rainstorm with 67.5 mm in early April 2012. Both graphs are based on data measured in the fairy circle F15 (bare patch and matrix). The complete TDR measurements from this fairy circle covering the entire period from early 2008 until 2022 are presented in [9]). (Online version in colour.)

By contrast, the fairy circles occur in an arid to hyperarid environment where the termites have to rely on extremely unpredictable, scant pulses of moisture received in rare rainfall events. Soil moisture measurements from 2008 to 2022 with TDR sensors (Decagon EC-5) at four different soil depths were sampled every 60 min at nine localities in Angola, Namibia and South Africa, to compare the soil moisture content within the fairy circles' bare patch with soil in the surrounding matrix vegetation [9]. Here and in figure 2, we present data from the fairy circle F15 (F15BP = bare patch and F15MT = matrix) at Dieprivier, Namibia. (During 15 years of continuous monitoring, fewer than 10 rainfall events occurred that were large enough to moisten the soil at 90 cm depth; for methods please refer to [10], chapter Methods, Table M1). In the following, we will show that the plants and the *Psammotermes* termites have apparently evolved very different strategies to enable them to make use of these rare and unpredictable pulses of moisture that are stored at relatively shallow depth in the soil.

Unlike the tall, above-ground mounds constructed by colonies of *Macrotermes*, colonies of sand termites do not construct any conspicuous above-ground structure. Consequently, there is typically no obvious indication of the local presence of *Psammotermes,* even when an active subterranean colony is present. Instead, a roughly circular bare patch within the vegetation, the fairy circle, is the most obvious visual feature indicating a colony's presence (figure 1). The term ‘fairy circle’ refers to the bare patch, and the term ‘fairy ring’ refers to the conspicuous belt of tall, long-lived grass tussocks (in the following called ‘perennial belt’) that often develops at the margin of the bare patch [14].

The permeable sandy soil in environments occupied by fairy circles allows rapid infiltration and percolation in the soil of both the bare patch and the surrounding vegetation (matrix). However, water loss through plant transpiration rapidly depletes soil water resources in areas surrounding the fairy rings (the matrix), but soil moisture is conserved within fairy rings due to the absence of transpiration. Within a few weeks after a strong rainfall that triggered plant germination, soil moisture has been markedly reduced through transpiration in the matrix, while the soil beneath the bare area remains very moist [14], fig. 2.

Soil of the bare patch does experience water losses by direct evaporation. However, evaporation losses of deeper soil levels become negligible once the upper 20–30 cm have become dry. In such a situation, effective evaporation of soil moisture takes place at the lower boundary of the upper dry layer and water losses depend on relatively slow, direct vapour diffusion through the sand [9].

The soil water content beneath the bare patch is higher than in the soil beneath the surrounding matrix at any given time. Evidence for this spatial distribution was gained from moisture measurements along the walls of trenches through fairy circles [10] and 15 years of measurement with time domain reflectometry (TDR) sensors at four different soil depths. Gröngröft & Jürgens [9] modelled the loss of stored soil moisture in relation to measured soil hydrological properties. They found that horizontal water loss of a moist bare patch to the surrounding dry matrix due to capillary water movement is controlled by the hydrological properties of the soil material and the diameter of the bare patch. The potential capillary water movement in sandy soils is rather low when most of the soil pores are filled with air and the connectivity of water-filled, medium-sized pores no longer exists. This occurs when the soil moisture levels are below field moisture capacity, which occurs once the surplus of infiltrated water has infiltrated to deeper sand layers due to the force of gravity. As a result, there is very little horizontal movement of soil moisture stored beneath bare patches to the drier soils of the surrounding matrix. With increasing diameter of the bare patch, water storage within the patch improves. Small bare patches are thus the first ones that lose their water reservoir through movement of capillary water. However, as calculations have revealed [9] in pure sands, even in a bare patch of only 3 m in diameter, the moisture may be stored for the duration of a dry season.

In addition to the above evidence for the course of soil moisture, the processes of germination, growth and dieback of the grasses within a fairy circle are also shown in Jürgens ([10], ch. 4), accompanied by a few selected photo series that capture the most important phases. Here, we present improved evidence in the form of five time-lapse videos of quite differing lengths.

This first WebCam video shows the germination and death of grasses within fairy circle number E91 in the Giribesvlakte, which was triggered by a rainfall of 32 mm on 12 February 2021. Germination is visible on day 5 after the rain (17 February 2021), first soil dumps are formed on day 13 (25 February 2021), first grasses die on day 26 (10 March 2021) and from day 31 (16 March 2021) onwards most grasses along three visible alleys of soil dumps (three subterranean tunnel systems) are dead or dying.

This second WebCam video shows the same processes within fairy circle F99 at Dieprivier, which was triggered by a rain of 14 mm on 02 January 2021. Germination is visible on day 4 after the rain (06 January 2021), best performance of grass plants is reached on day 28 (30 January 2021), first grasses die on day 30 (01 February 2021) and from day 36 (07 February 2021) onwards most grasses in the bare patch centre are dead or dying. On day 51 (22 February 2021) all grasses in bare patch are dead. Three more time-lapse videos up to a length of 2245 days are presented in the electronic supplementary material.

In summary, precipitation events of more than 10–12 mm trigger germination of the seedbank within fairy circles, but newly recruited, young plants rapidly disappear. From day 13 onwards, damage by *Psammotermes* termites and compacted sand at roots were observed [10] and around 31 days after the rain the above-ground parts of the grasses die and no longer transpire water.

The grasses first die in the centre of the bare patch; one to two weeks later the grasses at the margin also wilt. During this stage, the soil moisture beneath the bare patch persists at well above 5% water content in the range of grass roots, even at a depth of 30 cm below the soil surface (figure 2).

While the grasses in the bare patch quickly die because of herbivory by the termites, the grasses of the matrix vegetation typically remain alive during this phase of high soil moisture. In a year with high amounts of rainfall like 2011 (figure 2*b*), the soil beneath the matrix vegetation is still moist enough to allow continuous growth of the grasses for several months. In years with less rainfall, only one to two months may elapse before the matrix soil is too dry to support actively growing grasses (for example June 2012, figure 2*b*). However, the grass in the matrix becomes dormant and remains able to re-sprout if rain recurs within a couple of weeks or months. Without additional precipitation, though, the grasses will die, but the dry remains will persist.

The enhanced growth of tall grass tussocks within the perennial belt (fairy ring, luxury belt) is interpreted as a consequence of their privileged access to the water accumulation beneath the bare patch and the unidirectional suppression of competition, both caused by the termites and their behaviour [14]. In response to years with higher precipitation up to four additional perennial belts can be formed around bare patches with smaller diameters [10].

It is difficult to assess in detail the subterranean spatial distribution of the presence of the sand termite colony beneath a fairy circle. Excavations of the tunnel system are impossible due to the fragile nature of the sandy walls. However, the density of the portals of the tunnel system at the soil surface indicates the concentration of termite activity directly below the bare area. Termites expel sandy material waste from their tunnel systems through these portals. Such expulsion takes place in the early morning hours and creates small, moistened soil dumps that remain visible for a couple of hours. A map of the sand dump distribution typically shows highest densities (i.e. up to 90 portals/m^2^) in the bare patch centre, but remains in the range between 5 and 20 portals/m² in the matrix [14].

## 4. Colony and nest structure of *Psammotermes*

**Figure 3.**
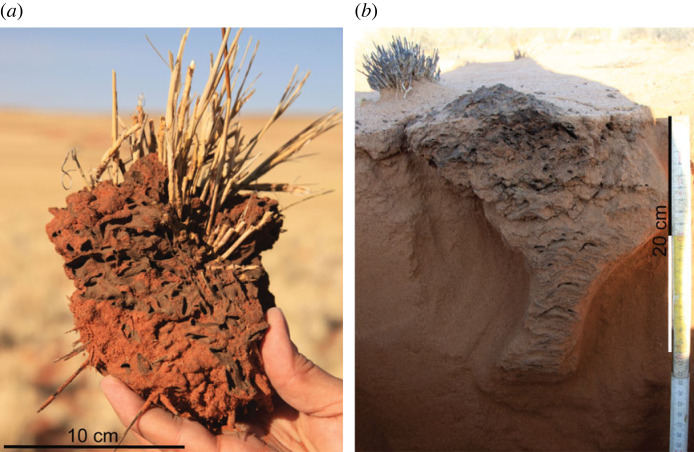
*Psammotermes* nest types. (*a*) Foraging nest, (*b*) permanent nest [14]. (Online version in colour.)

Two different types of nests have been observed in colonies of *Psammotermes*. Nests were first described by Coaton & Sheasby [53,54]. In habitats without wood, ‘nests of their own construction situated independently in the soil’ are ‘invariably centred in and below grass stools … and extended downwards into the sand to depths of 15–23 cm with the grass roots serving to bind together a rather brittle structure’ ([53], p. 57). Coaton & Sheasby [53,54] also describe galleries ‘no thicker than a matchstick’ and a ‘dark faecal lining of cells and passages'. Jürgens [14] calls this type of nest ‘foraging nest’ because he interprets these nests as a temporal structure used to consume one grass tussock. A typical foraging nest is shown in figure 3*a*.

Coaton & Sheasby [54] also noted a rarely found second type of nest, not associated with grass stools. These are described as ‘well-consolidated structures of dark carton ranging up to 36 cm and 30 cm in diameter and depth, respectively’ ([54], p. 26). Jürgens [14] calls this type of nest ‘permanent nest’ because he interprets these nests as a structure used over several years. A typical permanent nest is shown in figure 3*b*. From our studies after 2013, we can add that the depth of permanent nests below the soil surface sometimes exceeds 50 cm and may sometimes even reach 80 cm. We confirm that the permanent nests are less brittle than foraging nests because the faecal lining, called tapetum by Jürgens [14], cements the sand. Each foraging nest is connected to a system of mostly horizontal tunnels that enable the termites to move between the moist soil environment beneath the bare patch of the fairy circle and the dry environment, around the foraging nest ([10,14] fig. 5.7).

*Psammotermes* does not store larger amounts of food in underground chambers, but instead relies on regular harvest of material from the standing biomass in the matrix [14]. Most of the foraging nests are used only for a couple of weeks or months and are abandoned once most of the biomass of the tussock has been consumed.

**Figure 4.**
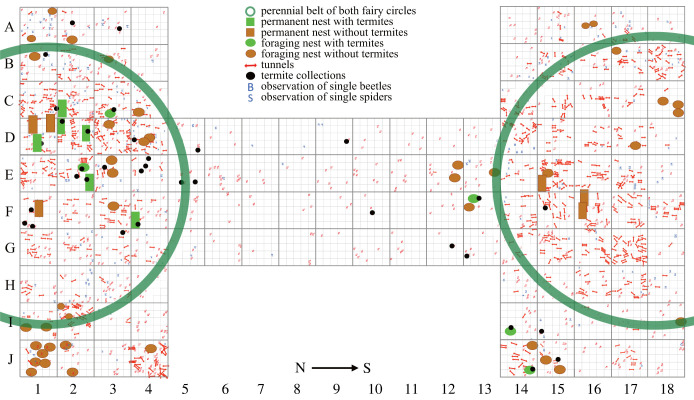
Map of nests, termites and tunnel elements found during a careful excavation of the top 30 cm below the surface (Giribesvlakte, Namibia, plot 36 167 (fairy circle left), plot 36 168 (fairy circle right), 06 March 2018). Dark green circular boundaries, perennial belts of both fairy circles; green squares, permanent nests with termites; ochre squares, permanent nests without termites; small bright green circles, foraging nests with termites; ochre circles, foraging nests without termites; red arrows, tunnels; black circles, termite collections; the much smaller blue symbols represent observations of single beetles or spiders without further relevance for the topic of this article. One large square (e.g. J1) is equal to 1 m^2^. (Online version in colour.)

As Coaton & Sheasby [54] noted, the permanent nests are less often found. This may partly be due to the fact that the foraging nests within grass tussocks are often elevated 1–3 cm above the soil surface, but the upper parts of the older permanent nests are frequently eroded due to exposure at the surface. Jürgens [14] found permanent nests at the perennial belt and in the central area of the bare patch and proposes that few of the foraging nests are developed further and become permanent nests at a later stage in their life history. Partial excavation of two neighbouring fairy circles (figure 4) revealed several permanent nests in the central area and near the half radius within the bare patch. This is the area where the termite colony shows highest activity, as measured by the density of ejection portals at the surface, near the area of the highest soil moisture content at the centre of the bare patch [14].

Several times we observed alates (reproductive potential queens and kings with wings) located in tunnels of permanent nests as well as separate tunnel systems, where they were apparently waiting for the first significant rainfall, which triggers their dispersal. To date, we have found royal chambers only in permanent nests of the Succulent Karoo group, while in the summer rainfall region to the north, royal chambers in permanent nests have not yet been collected or examined, despite excavations to depths of 2 m in search of these structures.

The short-lived foraging nests occur in the matrix, the perennial belt and the bare patch [14], as confirmed by the more recent excavation shown in figure 4. The spatial pattern of foraging nests can differ strongly in different years. In young fairy circles the majority of foraging nests are observed within the bare patch. In phases of annual growth of the fairy circles numerous foraging nests are found at the inner margin of the perennial belt [14]. In fully established fairy circles most foraging nests occur in the matrix. In 2023, a year without a single rainfall at Dieprivier, we found foraging nests exclusively within the dead perennial belts of the preceding years.

We propose that the spatial pattern of the foraging nests mainly reflects the patterns of the available food. The pattern of the permanent nests may simply reflect that a few of the foraging nests that formed during the establishment of a new fairy circle are persistently used and finally become permanent nests.

Our recent study of the genetic similarity patterns [39] confirms that the termites found in one fairy circle belong to one colony. All nests of different types in one sand termite colony are interconnected by a subterranean tunnel system ([10], fig. 5.7; [14]) that allows movements of the termites within and around one fairy circle. The termite colony of one fairy circle occupies more than one nest while some other nests remain unused [14], therefore the nest system should be classified as a polydomous or polycalic nest system [55].

Polydomous (or polycalic) nest systems are often found among primitive groups of subterranean termites including Hodotermitidae and Rhinotermitidae, of which *Psammotermes* is a member [56]. Polydomy is often the architectural solution to allow the presence of functional reproductives in more than one nest [57,58] as a precondition for budding. This is not the case in *Psammotermes*. Genetic analyses of individuals from clusters of neighbouring fairy circles at several fairy circle landscapes revealed no indication of budding but genetically distinct populations within each fairy circle [39].

At this point, we would like to explore whether, based on the earlier published knowledge or new evidence shown here, the polydomous colony structure can be interpreted in the context of the extremely limited soil moisture conditions in the soil of the matrix vegetation and the stored moisture beneath the bare patch.

Colonies of many termite taxa establish large, above-ground (epigeal) mounds that house their centrally located nests. Thick and solidly cemented mounds of some species provide defence against predators and allow an extended manipulation of the hydrological, atmospheric and chemical environment [50,59–61].

We propose that the construction of large mounds and accompanying central nests cannot be expected in extremely arid environments because of the scarcity of resources. In the Namib, the small mounds of *Baucaliotermes hainsii* are found in climates with more than 100 mm MAP, and larger mounds of *Macrotermes* species well above 200 mm MAP (N Juergens, F Gunter 2022, personal observations; [40,62]).

*Psammotermes* colonies in the desert typically have a population size of only a few hundred to one thousand individuals (N Juergens, F Gunter 2022, personal observations). Even in moister environments of the Namib Desert region, for example, the gallery forest of the Kuiseb River at Gobabeb, no colonies of more than 2800 individuals were found [63].

It may be crucial to interpret the colony structure from a perspective that focuses on the key innovation: the storage of rainwater beneath the bare patch. While the bareness of the bare patch enables the storage of water and elimination of water extraction by plants, it also requires termites to forage in the matrix vegetation with its dry soil. The construction of a sheltering, temporary foraging nest directly within the basal parts of the consumed grass tussock may well be an adequate solution. Laboratory stress experiments indicate that single termite individuals in environments with relative humidities ranging from 33% to 93% survive for a corresponding range of only 5–19 h [63]. Consequently, these conditions require either a frequent movement of the termites between the two essential resources or a frequent transport of water to the foraging nests within the termite bodies.

**Figure 5.**
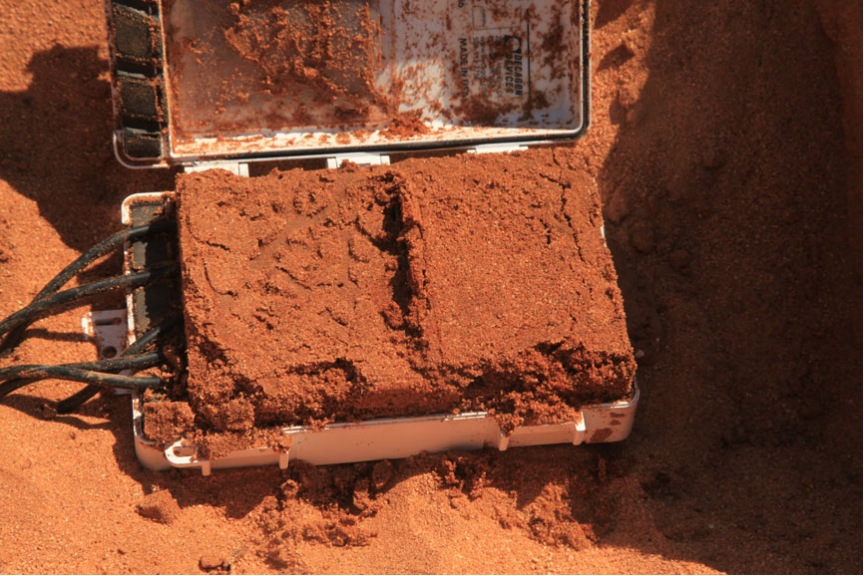
A logger for storage of the soil moisture data located in dry sand at 10 cm below the soil surface is found filled with very moist sand brought in by *Psammotermes* termites (Giribesvlakte, Namibia, 19 May 2015). (Online version in colour.)

We do not know to what extent termites are able to transport water for purposes beyond their own water consumption. However, frequently the containers of data loggers used in measurement of soil moisture became occupied by sand termites and were filled with moist sand (figure 5). This observation clearly indicates the occurrence of active water transport by termites.

At first glance, the lack of a central nest seems to contradict the fact that the termite colony shows highest activity, as measured by the density of ejection portals at the surface, near the area of the highest soil moisture content at the centre of the bare patch [14]. However, the location of the permanent nests at the margin of the bare patch has the advantage of a short distance to both the food and the water.

It should also be considered whether polydomy may also serve to lessen the likelihood of destruction of a colony by predators. As has been shown earlier [14,64], the fairy circles attract a rich satellite fauna, including predators with various positions in rich food webs that are based on the perennial presence of *Psammotermes* termites (in combination with the storage of rain water in the soil and in the termite bodies). Henschel & Jürgens [64] report on numerous mammals, reptiles, spiders (including spiders of the family Ammoxenidae specialized to hunt *Psammotermes*) and several insect groups. It is important that, especially in the extremely arid parts of the Namib and during series of drought years, perennial life in the desert is only possible because of the perennial provision of water and food by the fairy circles. In particular, the destructive larger mammals like *Orycteropus afer* (aardvark) or *Prosteles cristata* (Aardwolf) could easily extinguish a termite colony that consists of one nest only.

## 5. Towards extreme aridity: progression of evolutionary steps

Historically, all sand termite collections in the arid regions of southern Africa described in [54] were regarded as one species. However, recent morphological and molecular studies with a focus on the sand termite populations of the more arid regions in southern Africa revealed a complex of eight well-supported species [38,65].

Beyond the cladogram already published in [38,65] and its taxonomic relevance, the reconstruction of the evolutionary history allows us to discuss here the sequence of newly developed properties that may be related to the capacity to live in hyperarid environments.

**Figure 6.**
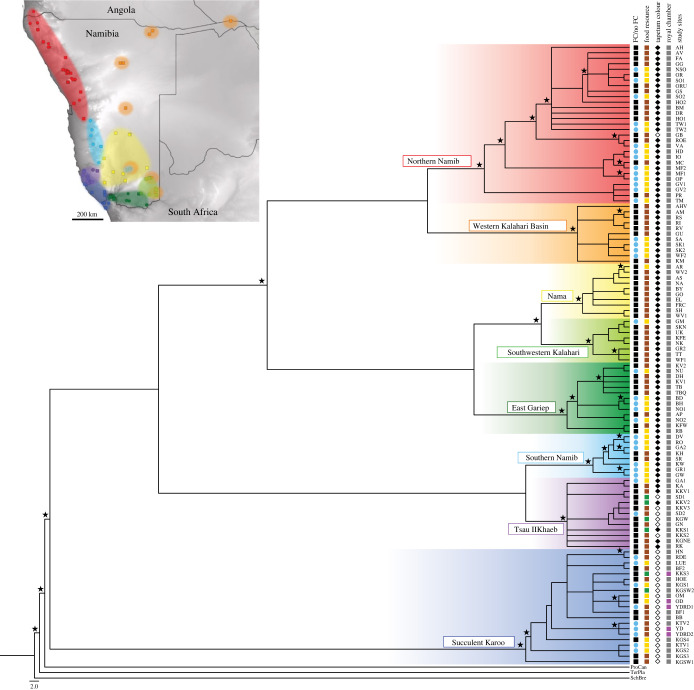
Phylogeny and distribution of the eight distinct species of the *Psammotermes allocerus* aggregate comprising 113 study sites and gained from COI and COII markers adapted to [38]. Posterior probability values above 0.98 are highlighted with a star above the branches. Clades are coloured according to their genetic group [38]. Symbols for the traits: collection site: within fairy circle (FC) = blue circle, not in a fairy circle region (no FC) = black square; food resource: wood = brown square, grass = yellow square, *Euphorbia gummifera* = green square; Tapetum colour: blackish = black diamond, whitish = white diamond; nest with a royal chamber between 15 and 30 cm below the surface: royal chamber = pink square, no royal chamber = grey square. Abbreviations of the study sites are listed in the electronic supplementary material. (The figure of the phylogenetic tree is a permitted copy from Gunter *et al*. [38]. The presentation of the states of five traits for each single sample is presented here for the first time). (Online version in colour.)

As shown in figure 6, the most basal clade of this complex is located in the Succulent Karoo (dark blue) [38,65] within the winter rainfall regime of southern Africa. The formation of fairy circles by this species is only observed at localities with a MAP between 50 and 80 mm, i.e. in the Atlantic coastal region north of Port Nolloth, located at the coast at 29,25° S latitude. This region additionally receives significant inputs of moisture from fog. Within this area, colonies of *Psammotermes* (sp. nov. ‘Succulent Karoo’) occupying bare patches forage on roots and stems of the dominant plant species, i.e. mostly Aizoaceae, Geraniaceae and Euphorbiaceae, as well as the grasses *Stipagrostis geminifolia* and *S. ciliata* [1]. In addition, tall shrubs of *Euphorbia gummifera* often support colonies of *Psammotermes* with a large columnar nest established within the phytogenic dune in lee of the shrub (figure 6, food resource column). Only within this species, royal chambers with a termite king and queen in fairy circles were found in nests 15–30 cm below the soil surface ([8], figure 6, royal chamber column). In all other fairy circle localities of the *Psammotermes allocerus* aggregate, no royal chamber has yet been found, probably because of deeper positioning (greater than 1 m depth; figure 6, royal chamber column).

After *Psammotermes* sp. nov. ‘Succulent Karoo’, the next step within the phylogenetic tree *Psammotermes allocerus* goes along with the occupation of areas with tropical summer rainfall climate. This clade is subdivided into two subclades: *Psammotermes* sp. nov. ‘Tsau//Khaeb’ (purple) and *Psammotermes* sp. nov. ‘Southern Namib’ (bright blue).

*Psammotermes* sp. nov. ‘Tsau//Khaeb’ (purple) inhabits the transition between winter and summer rainfall in the northern parts of the Tsau//Khaeb National Park in southwestern Namibia. This taxon forages on the roots and stems of *Euphorbia gummifera* as main food source, as already described for some populations of *Psammotermes* sp. nov. ‘Succulent Karoo’, sometimes combined with fairy circles formed in areas occupied by grasses.

*Psammotermes* sp. nov. ‘Southern Namib’ (bright blue) creates fairy circles in the dunes at the eastern margin of the Namib Sand Sea and is phylogenetically the first species that almost solely forages on grasses. Only two populations were found foraging dead wood in areas without fairy circle appearance (KH, Keetmanshoop and SR, Sesriem, figure 6).

All five clades higher up in the phylogenetic tree are sister to the previous clades and inhabit regions either further east or further north.

A more eastern group comprises the three taxa *Psammotermes* sp. nov. ‘East Gariep’ (dark green), *Psammotermes* sp. nov. ‘Southwestern Kalahari’ (light green) and *Psammotermes* sp. nov. ‘Nama’ (yellow). For these three taxa, only six fairy circle sites out of 31 sand termite observations are documented for East Gariep and Southeastern Kalahari. In these regions, *Psammotermes* consume wood and grass.

The more northern group splits into a more arid and a more humid sister clade. The more arid clade *Psammotermes* sp. nov. ‘Northern Namib’ (red) inhabits the northern Namib Desert of Namibia and SW Angola (red in figure 6). Wherever the sand termite feeds on grasses and occurs in permeable, sandy soils it creates fairy circles (e.g. *Psammotermes* sp. nov. ‘Northern Namib’ or *Psammotermes* sp. nov. ‘Southern Namib’). On sandy soils in riverine forest without fairy circle formation, wood is the main food resource.

Termites of the more eastern clade *Psammotermes* sp. nov. ‘Western Kalahari Basin’ (ochre) inhabit the Savanna Biome and mostly feed on wood (e.g. in the study site KM, AHV) while in the arid southeast of Namibia they create fairy circles by feeding on grasses in sandy soils (e.g. in the study site SK, SA).

The colour of the inside nest wall (called tapetum) differs between the taxa of this complex. The tapetum wall of all excavated nests of the Succulent Karoo is whitish [38]. This was also documented for six out of 13 nests within the Tsau //Khaeb taxon and for one nest at Gobabeb (GB) within the Northern Namib taxon (figure 6). The tapetum of all other nests is blackish, regardless of the food resource (wood, grass or *E. gummifera*, figure 6). At present, these differences are only distinguishing characters, without evidence regarding causation or function.

The sand termite also occurs in more humid regions of Southern Africa as described in [54], which would probably be positioned basally in the phylogeny. Only few detailed descriptions on occurrences of sand termite nests in more humid regions (Botswana, southeastern Africa) are available. In these regions, the sand termite is known as a pest that uses timber from buildings as a food source, but also growing plants and grain crops [54].

## 6. Discussion: sand termite behaviour: an adaptation to extreme aridity?

The features of sand termite colonies described above can be understood as a successful adaptation to the conditions of an extremely arid ecosystem: the zonation of vegetation as triggered by localized herbivory, the capture and storage of rain water beneath the bare patch and the diversified polydomous nest and tunnel system.

A) The key adaptation aims at the most important resource in an arid system: water. The termites manipulate the availability of soil moisture by removal of the transpiring plants within the bare patch. Through this process, the termites ensure the conservation of soil water required for colony survival by manipulation of processes involved in the flux of moisture from the soil to the atmosphere. At the same time, they create small patches of perennial life in the desert that also allow permanent survival of some plants and of a rich food network of predators next to the conserved soil moisture [14].
B) As a consequence of the above-described way in which the termites control the availability of water, the foraging must be spatially separated (in spite of the small size of the colony) and principally takes place in the surrounding matrix vegetation and, only during certain periods, in the perennial belt. This spatial separation of the function of water storage beneath the bare patch and of foraging outside of that patch requires frequent movements of the termites between these two key resources.
C) The need for these two resources—soil moisture and plant foods—has apparently driven the evolution of two different types of nest construction by colonies. One of the two types, the foraging nest, is not involved in reproduction, but rather enables efficient, locally focused foraging of grass biomass even during months of extreme arid soil conditions. The other type, the permanent nest, serves the function of improved long-term protection, reproduction, and possibly a minor level of food storage [14].
D) Due to the spatial separation of the functions of water storage in soil of the bare patch and of foraging outside of that patch, no accumulation of nutrients (e.g. N, P, K) occurs in the centre of the fairy circle. Similarly, the only very small size of biomass storage in combination with digestion of grass tussocks in the tussock itself also minimizes the transport of biomass and nutrients. While other termites accumulate large amounts of biomass within a central nest [41,43] or even strongly alter the chemical environment in the centre of the colony [66], we propose that in an extreme desert it is more important to be a hydrological ecosystem engineer than a chemical ecosystem engineer.
E) The ability of the fairy circle to store rainwater over extended periods of drought may well have enabled the organism *Psammotermes allocerus* to inhabit one of the more extreme desert environments on earth. A second driver of evolution may have been the ability to escape a wide array of predators that cannot survive the extreme conditions of the desert, especially in deep, loose sands.

## Data Availability

The data are provided in electronic supplementary material [67].
